# Supplementary material for: The Association between Acute and Late Genitourinary and Gastrointestinal Toxicities: An Analysis of the PACE B Study
Source: Cancers (Basel). 2023 Feb 17;15(4):1288. doi: 10.3390/cancers15041288 (PMC9954684; doi:10.3390/cancers15041288)
Supplement: Supplementary file 1 [file cancers-2201262-supplementary.pdf › cancers-2201262-supplementary.pdf]

Supplementary Table S1. CTCAE Assessment Completion Rates

CTCAE assessment	Per Protocol Treatment SBRT				Per Protocol treatment CRT			
	GI toxicity		GU toxicity		GI toxicity		GU toxicity	
	No.	%	No.	%	No.	%	No.	%
**Baseline**								
Assessed	413	99	412	99	430	99	431	99
Missing	2	1	2	1	4	1	3	1
**End of treatment (SBRT only)**								
Assessed	398	97	399	97	-	-	-	-
Missing	14	3	13	3	-	-	-	-
**Week 2 Post-RT**								
Assessed	390	94	390	94	389	90	389	90
Missing	25	6	25	6	45	10	45	10
**Week 4 Post-RT**								
Assessed	403	97	400	96	411	95	411	95
Missing	12	3	15	4	23	5	23	5
**Week 8 Post-RT**								
Assessed	374	90	374	90	393	91	391	90
Missing	41	10	41	10	41	9	43	10
**Week 12 Post-RT**								
Assessed	405	98	405	98	421	97	421	97
Missing	10	2	10	2	13	3	13	3
**6 months Post-RT**								
Assessed	393	95	394	95	422	97	422	98
Missing	21	5	20	5	11	3	11	2
**9 months Post-RT**								
Assessed	392	95	393	95	410	95	412	96
Missing	21	5	20	5	21	5	19	4
**12 Months Post-RT**								
Assessed	405	98	404	98	413	96	413	96
Missing	7	2	8	2	18	4	18	4
**15 months Post-RT**								
Assessed	320	78	321	78	328	76	338	76
Missing	92	22	91	22	102	24	102	24
**18 months Post-RT**								
Assessed	376	92	329	92	393	91	392	91
Missing	33	8	34	8	37	9	38	9
**21 months Post-RT**								
Assessed	318	78	316	78	320	75	319	74
Missing	88	22	90	22	109	25	110	26
**24 months Post-RT**								
Assessed	380	96	379	96	378	92	376	91
Missing	17	4	18	4	34	8	36	9

Supplementary Table S2. EPIC-26 Assessment Completion Rates

EPIC-26 assessment	Per Protocol Treatment SBRT				Per Protocol treatment CRT			
	EPIC-26 overall urinary bother		EPIC-26 bowel sub-domain score		EPIC-26 overall urinary bother		EPIC-26 bowel sub-domain score	
	No.	%	No.	%	No.	%	No.	%
**Baseline**								
Assessed	384	93	365	88	404	93	390	90
Missing	31	7	50	12	30	7	44	10
**Week 4 Post-RT**								
Assessed	362	87	348	84	353	81	340	78
Missing	53	13	67	16	81	19	94	22
**Week 12 Post-RT**								
Assessed	382	92	366	88	386	89	364	84
Missing	33	8	49	12	48	11	70	16
**6 months Post-RT**								
Assessed	314	76	295	71	322	74	306	71
Missing	100	24	119	29	111	26	127	29
**9 months Post-RT**								
Assessed	303	73	290	70	315	73	300	70
Missing	110	27	123	30	116	27	131	30
**12 Months Post-RT**								
Assessed	376	91	351	85	377	87	347	81
Missing	36	9	61	15	54	13	84	19
**24 months Post-RT**								
Assessed	338	85	318	80	339	82	316	77
Missing	59	15	79	20	73	18	96	23

Supplementary Table S3. IPSS assessment Completion Rates

IPSS assessment	Per Protocol Treatment			
	SBRT		CRT	
	No.	%	No.	%
**Baseline**				
Assessed	358	86	379	87
Missing	57	14	55	13
**Week 2 Post-RT**				
Assessed	340	82	355	82
Missing	75	18	79	18
**Week 4 Post-RT**				
Assessed	345	83	332	76
Missing	70	17	102	24
**Week 8 Post-RT**				
Assessed	336	81	350	81
Missing	79	19	84	19
**Week 12 Post-RT**				
Assessed	362	87	373	86
Missing	53	13	61	14
**6 months Post-RT**				
Assessed	352	85	361	83
Missing	62	15	72	17
**9 months Post-RT**				
Assessed	351	85	360	83
Missing	62	15	74	17

**12 Months Post-RT**				
Assessed	365	89	365	85
Missing	47	11	66	15
**24 months Post-RT**				
Assessed	300	76	312	76
Missing	97	24	100	24

Supplementary Table S4. Patient level assessment completion rate

**Time Period**	**Number of CTCAE assessments completed**	**CRT**		**SBRT**	
		**CTCAE GU completion (%)**	**CTCAE GI completion (%)**	**CTCAE GU completion (%)**	**CTCAE GI completion (%)**
Acute	All*	79.4	80.4	80.4	80.9
	At least three	93.9	93.7	98.7	98.8
Late	All†	60.0	59.8	62.1	61.8
	At least five	92.2	92.3	92.0	92.2

*In acute period the maximum visits were four for the CRT population and five for the SBRT population
†In the late period the maximum visits were seven for all treatment populations. GU= genitourinary; GI=gastrointestinal.

Supplementary Table S5: Univariable and multivariable logistic regression model to identify covariates associated with CTCAE late genitourinary toxicity (12-24 months) after SBRT

Co-variate	Level	CTCAE grade 2+ GU late toxicity (12-24 months)					
		No (n=303)	Yes (n=108)	Univariable		Multivariable	
		N (%) or median (IQR)	N (%) or median (IQR)	OR (95% CI)	p value	OR (95% CI)	p value
Baseline grade 2+ GU toxicity	No<br>Yes	295 (98)<br>6 (2)	93 (86)<br>15 (14)	**7.93 (2.99-21.02)**	**<0.0001**	**4.33 (1.50-12.5)**	**0.007**
IPSS total score (baseline)	Median<br>(n)	5 (3-10)<br>(n=262)	8 (4-15)<br>(n=92)	**1.08 (1.04-1.12)**	**<0.0001**		
EPIC-26 overall urinary bother (baseline)	Median<br>(n)	100 (75-100)<br>(n=283)	75 (50-100)<br>(n=97)	**0.98 (0.97-0.99)**	**<0.0001**		
Acute grade 2+ GU Toxicity	No<br>Yes	234 (77)<br>69 (23)	48 (44)<br>60 (56)	**4.24 (2.66-6.75)**	**<0.0001**	**3.29 (1.98-5.44)**	**<0.0001**
Persistent grade 2+ acute GU toxicity	No<br>Yes	279 (92)<br>24 (8)	70 (65)<br>38 (35)	**6.31 (3.55-11.2)**	**<0.0001**		
IPSS total score (acute)	Median<br>(n)	12 (8-18)<br>(n=294)	17 (11-24)<br>(105)	**1.07 (1.04-1.11)**	**<0.0001**		
Lowest EPIC score overall urinary bother (acute)	Median<br>(n)	75 (50-100)<br>(n=296)	50 (25-75)<br>(n=102)	**0.98 (0.97-0.99)**	**<0.0001**		
CTCAE specific symptoms							
Grade 2+ obstructive	No<br>Yes	288 (95)<br>15 (5)	86 (80)<br>21 (20)	**4.69 (2.31-9.49)**	**<0.0001**		
Grade 2+ irritative	No<br>Yes	258 (85)<br>45 (15)	61 (57)<br>47 (43)	**4.42 (2.69-7.25)**	**<0.0001**		
Grade2+ pain/dysuria	No<br>Yes	279 (92)<br>24 (8)	88 (82)<br>19 (18)	**2.51 (1.31-4.80)**	**0.005**		
Grade 2+ incontinence	No<br>Yes	300 (99)<br>3 (1)	97 (91)<br>10 (9)	**10.31 (2.78-38.22)**	**<0.0001**		
Grade 1+ haematuria	No<br>Yes	276 (91)<br>27 (9)	94 (88)<br>13 (12)	1.41 (0.71-2.85)	0.334		
Age	Median<br>(n)	69 (65-73)<br>(n=303)	68.5 (66-73)<br>(n=108)	1.01 (0.98-1.05)	0.46		
Risk group (Intermediate)	low<br>intermediate	24 (8)<br>279 (92)	11 (10)<br>97 (90)	0.76 (0.36-1.61)	0.47	0.59 (0.26-1.36)	0.21
Prostate volume (cm^3)	Median<br>(n)	40 (31-55)<br>(n=299)	43 (34-62)<br>(n=107)	**1.01 (1.00-1.02)**	**0.02**	1.01 (1.00-1.02)	0.17
Urinary medication use (Yes)	No<br>Yes	244 (80)<br>59 (20)	80 (74)<br>28 (26)	1.45 (0.86-2.42)	0.16	1.23 (0.69-2.19)	0.49
SBRT technique (CL)	CyberKnife<br>CL	135 (45)<br>167 (55)	33 (31)<br>72 (67)	**1.76 (1.11-2.82)**	**0.018**	**2.12 (1.21-3.73)**	**0.009**
Fiducial use (Yes)	No<br>Yes	90 (30)<br>213 (70)	20 (18)<br>88 (82)	**1.86 (1.07-3.20)**	**0.03**	**2.62 (1.39-4.96)**	**0.003**

OR= odds ratio; IQR=interquartile range; CL= conventional linac; GU= genitourinary; IPSS = international Prostate Symptom Score; EPIC=Expanded Prostate Cancer Index Composite

Supplementary Table S6: Univariable and multivariable logistic regression model to identify covariates associated with CTCAE late genitourinary toxicity (12-24 months) after CRT

Co-variate	Level	CTCAE grade 2+ GU late toxicity (12-24 months)					
		**No (n=366)**	**Yes (n=59)**	**Univariable**		**Multivariable**	
		**N (%) or median (IQR)**	**N (%) or median (IQR)**	**OR (95% CI)**	**p value**	**OR (95% CI) (n-407)**	**p value**
Baseline grade 2+ GU toxicity	No<br>Yes	354 (98)<br>9 (2)	49 (83)<br>10 (17)	**8.03 (3.11-20.73)**	**<0.0001**	**5.40 (1.97-14.8)**	**0.001**
IPSS total score (baseline)	Median<br>(n)	6 (3-11)<br>(n=317)	8 (4-13)<br>(n=55)	1.04 (1.00-1.08)	0.08		
EPIC-26 overall urinary bother (baseline)	Median<br>(n)	100 (75-100)<br>(n=341)	75 (50-100)<br>(n=56)	**0.99 (0.98-1.00)**	**0.02**		
Acute grade 2+ GU Toxicity	No<br>Yes	291 (80)<br>75 (20)	34 (59)<br>24 (41)	**2.74 (1.53-4.90)**	**0.001**	**2.27 (1.22-4.22)**	**0.009**
Persistent grade 2+ acute GU toxicity	No<br>Yes	345 (94)<br>21 (6)	39 (66)<br>20 (34)	**8.42 (4.20-16.90)**	**<0.0001**		
IPSS total score (acute)	Median<br>(n)	12 (7-18)<br>(n=356)	15 (10-20)<br>(n=59)	**1.05 (1.02-1.09)**	**0.005**		
Lowest EPIC overall urinary bother (acute)	Median<br>(n)	75 (50-100)<br>(n=351)	50 (25-75)<br>(n=59)	**0.98 (0.97-0.99)**	**<0.0001**		
CTCAE specific symptoms							
Grade 2+ obstructive	No<br>Yes	349 (95)<br>17 (5)	45 (78)<br>13 (22)	**5.93 (2.70-13.0)**	**<0.0001**		
Grade 2+ irritative	No<br>Yes	310 (85)<br>56 (15)	44 (76)<br>14 (24)	1.76 (0.91-3.42)	0.10		
Grade 2+ pain/dysuria	No<br>Yes	350 (96)<br>16 (4)	56 (97)<br>2 (3)	0.78 (0.17-3.49)	0.75		
Grade2+ incontinence	No<br>Yes	360 (98)<br>6 (2)	55 (95)<br>3 (5)	3.27 (0.80-13.47)	0.10		
Grade1+ haematuria	No<br>Yes	353 (96)<br>13 (4)	56 (97)<br>2 (3)	0.97 (0.21-4.41)	0.97		
Age	Median<br>(n)	69 (65-74)<br>(n=366)	69 (66-73)<br>(n=59)	1.00 (0.96-1.05)	0.85		
Risk group (Intermediate)	low<br>intermediate	39 (11)<br>327 (89)	3 (5)<br>56 (95)	2.23 (0.67-7.45)	0.19	1.94 (0.56-6.68)	0.29
Prostate volume (cm^3)	Median<br>(n)	43 (33-56)<br>(n=352)	46 (33-65)<br>(n=58)	1.01 (1.00-1.02)	0.16		
Urinary medication use (Yes)	No<br>Yes	290 (79)<br>76 (21)	42 (71)<br>17 (29)	1.54 (0.83-2.86)	0.17		
Fiducial use (Yes)	No<br>Yes	155 (42)<br>211 (58)	28 (47)<br>31 (53)	0.81 (0.47-1.41)	0.46	1.08 (0.59-1.94)	0.81

OR= odds ratio; IQR=interquartile range ; GU= genitourinary; IPSS = international Prostate Symptom Score; EPIC=Expanded Prostate Cancer Index Composite

Supplementary Table S7: Univariable and multivariable logistic regression model to identify covariates associated with CTCAE late gastrointestinal toxicity (12-24 months) after SBRT

Covariates	Level	CTCAE grade 2+ GI late toxicity (12-24 months)					
		No (n=369)	Yes (n=42)	Univariable		Multivariable	
		N (%) or median (IQR)	N (%) or median (IQR)	OR (95% CI)	p value	OR (95% CI) (n=410)	p value
Baseline grade 2+ GI toxicity	No<br>Yes	363 (99)<br>4 (1)	42 (100)<br>0 (0)				
EPIC-26 bowel sub-domain score (baseline)	Median (n)	100 (92-100)<br>(n=323)	96 (87.5-100)<br>(n=38)	**0.96 (0.93-0.99)**	**0.006**		
Worst acute grade 2+ GI Toxicity	No<br>Yes	318 (86)<br>51 (14)	29 (69)<br>13 (31)	**2.80 (1.36-5.73)**	**0.005**	**2.90 (1.40-6.03)**	**0.004**
Persistent grade 2+ acute GI toxicity	No<br>Yes	359 (97)<br>10 (3)	38 (90)<br>4 (10)	**3.78 (1.13-12.63)**	**0.031**		
Worst EPIC-26 bowel sub-domain score (acute)	Median (n)	87.5 (75-95.8)<br>(n=356)	79.2 (62.5-91.7)<br>(n=40)	**0.98(0.97-1.00)**	**0.02**		
CTCAE specific acute symptoms							
Grade 1+ rectal bleeding	No<br>Yes	280 (76)<br>88 (24)	26 (62)<br>16 (38)	1.96 (1.00-3.82)	0.05		
Grade 2+ bowel frequency	No<br>Yes	346 (94)<br>22 (6)	37 (88)<br>5 (12)	2.13 (0.76-5.94)	0.15		
Grade 1+ rectal pain	No<br>Yes	278 (75)<br>90 (25)	20 (48)<br>22 (52)	**3.40 (1.77-6.51)**	**<0.0001**		
Grade 2+ proctitis	No<br>Yes	352 (95)<br>17 (5)	36 (86)<br>6 (14)	**3.45 (1.28-9.30)**	**0.01**		
Age	Median (n)	69 (65-73)<br>(n=369)	69 (64-73)<br>(n=42)	1.00 (0.95-1.05)	0.95		
Prostate Volume (cm^3)	Median (n)	40 (31-56)<br>(n=365)	40 (34-63)<br>(n=41)	1.01 (0.99-1.02)	0.22		
Risk group (intermediate)	low<br>Int	30 (8)<br>339 (92)	5 (12)<br>37 (88)	0.65 (0.24-1.79)	0.41		
SBRT modality (CL)	CyberKnife<br>CL	157 (43)<br>210 (57)	11 (26)<br>29 (69)	1.97 (0.55-4.01)	0.07	1.67 (0.73-3.86)	0.225
Fiducial use (Yes)	No<br>Yes	95 (26)<br>274 (74)	15 (36)<br>27 (64)	0.62 (0.32-1.22)	0.17	0.77 (0.35-1.69)	0.511

OR= odds ratio; IQR=interquartile range; CL= conventional linac; GI= gastrointestinal; EPIC=Expanded Prostate Cancer Index Composite; Int= intermediate.

Supplementary Table S8: Univariable and multivariable logistic regression model to identify covariates associated with CTCAE late gastrointestinal toxicity (12-24 months) after CRT

Covariates	level	CTCAE grade 2+ GI late toxicity (12-24 months)			
		No (n=383)	Yes (n=42)	Univariable	
		N (%) or median (IQR)	N (%) or median (IQR)	OR (95% CI)	p value
Baseline grade 2+ GI toxicity	No<br>Yes	378 (99)<br>3 (1)	40 (100)<br>0 (0)		
EPIC-26 bowel sub-domain score (baseline)	Median<br>(n)	100 (96-100)<br>(n=346)	100 (92-100)<br>(n=37)	0.98 (0.93-1.04)	0.55
Worst acute grade 2+ GI Toxicity	No<br>Yes	353 (92)<br>29 (8)	35 (83)<br>7 (17)	2.43 (0.99-5.96)	0.051
Persistent grade 2+ acute GI toxicity	No<br>Yes	379 (99)<br>4 (1)	39 (93)<br>3 (7)	**7.29 (1.57-33.75)**	**0.011**
Worst EPIC-26 bowel bother (acute)	Median<br>(n)	92 (75-100)<br>(n=360)	88 (71-96)<br>(n=41)	0.98 (0.96-1.00)	0.09
CTCAE specific acute symptoms					
Grade 1+ rectal bleeding	No<br>Yes	324 (85)<br>58 (15)	32 (76)<br>10 (24)	1.75 (0.81-3.74)	0.15
Grade 2+ bowel frequency	No<br>Yes	376 (98)<br>6 (2)	42 (100)<br>0 (0)		
Grade 1+ rectal pain	No<br>Yes	335 (88)<br>47 (12)	33 (79)<br>9 (21)	1.94 (0.88-4.31)	0.10
Grade 2+ proctitis	No<br>Yes	373 (98)<br>9 (2)	40 (95)<br>2 (5)	2.07 (0.43-9.93)	0.36
Age	Median<br>(n)	69 (65-73)<br>(n=383)	69.5 (66-74)<br>(n=42)	1.02 (0.97-1.07)	0.48
Prostate Volume	Median<br>(n)	44 (33-58)<br>(n=373)	43 (32-51)<br>(n=37)	0.99 (0.98-1.01)	0.50
Risk group (intermediate)	low<br>Int	42 (11)<br>341 (89)	0 (0)<br>42 (100)		
Fiducial use (Yes)	No<br>Yes	164 (43)<br>219 (57)	19 (45)<br>23 (55)	0.91 (0.48-1.72)	0.76

OR= odds ratio; IQR=interquartile range;; GI= gastrointestinal; EPIC=Expanded Prostate Cancer Index Composite; Int = intermediate.

Supplementary Table S9: Bladder dose volume parameters univariable analysis

Covariate	Univariable Analysis (acute G2+ events)			Univariable analysis (late G2+ event)		
	OR (95% CI)	n	p value	OR (95% CI)	n	p value
Bladder dose volume parameters						
Bladder V18.1Gy (<40%)	1.01 (1.00-1.03)	413	0.08	0.99 (0.97-1.01)	412	0.55
Bladder V37Gy (<5cc/10cc)	1.00 (0.95-1.07)	413	0.20	1.04 (0.98-1.10)	412	0.22

Supplementary Table S10: Rectal dose volume parameters univariable analysis

Covariate	Univariable Analysis (acute G2+ events)			Univariable analysis (late G2+ event)		
	OR (95% CI)	n	p value	OR (95% CI)	n	p value
Rectal dose volume parameters						
Rectum V18.1Gy (<50%)	1.01 (0.98-1.03)	413	0.56	1.00 (0.98-1.03)	412	0.61
Rectum V29Gy (<20%)	1.04 (0.99-1.09)	413	0.13	1.03 (0.97-1.08)	412	0.35
Rectum V36Gy (<1cc)	1.11 (0.89-1.39)	413	0.37	1.00 (0.74-1.36)	412	0.98

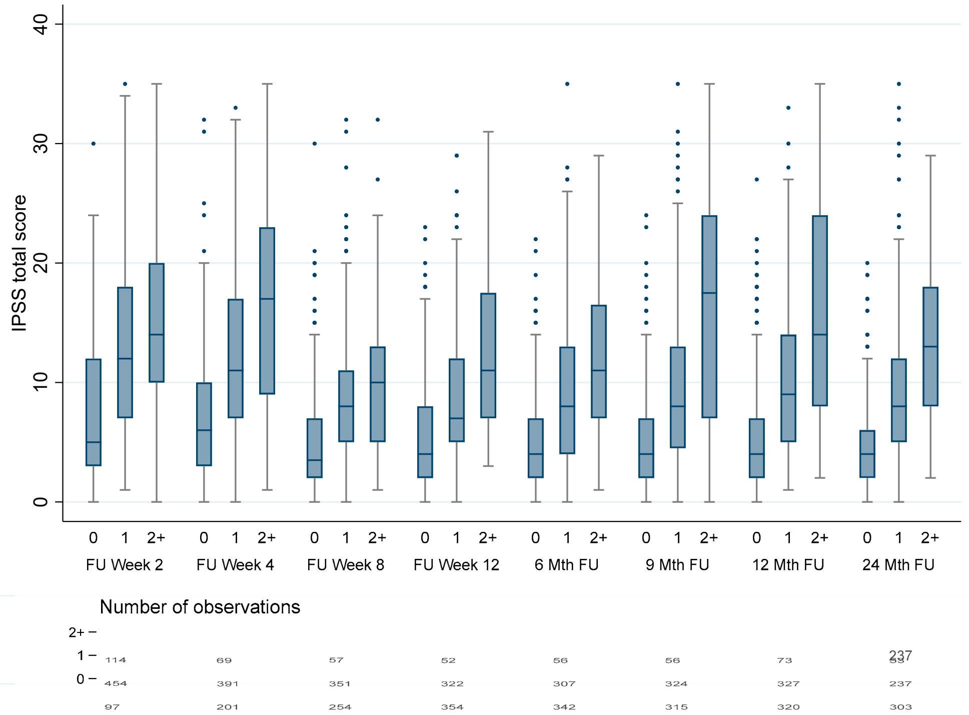

Figure S1: Association between IPSS total score and CTCAE GU grading across acute and late time periods (x axis). IPSS score 0-7 = mild, 8-19 =moderate, 20-35=severe.

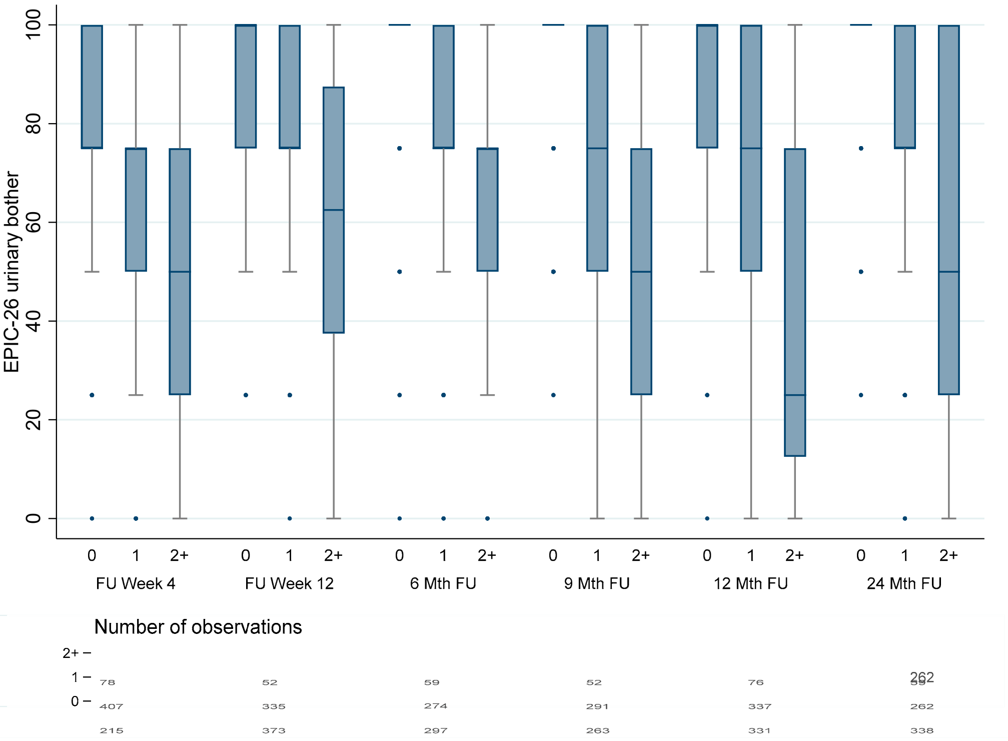

Figure S2: Association between EPIC-26 urinary bother and CTCAE GU grading across acute and late time periods (x axis).

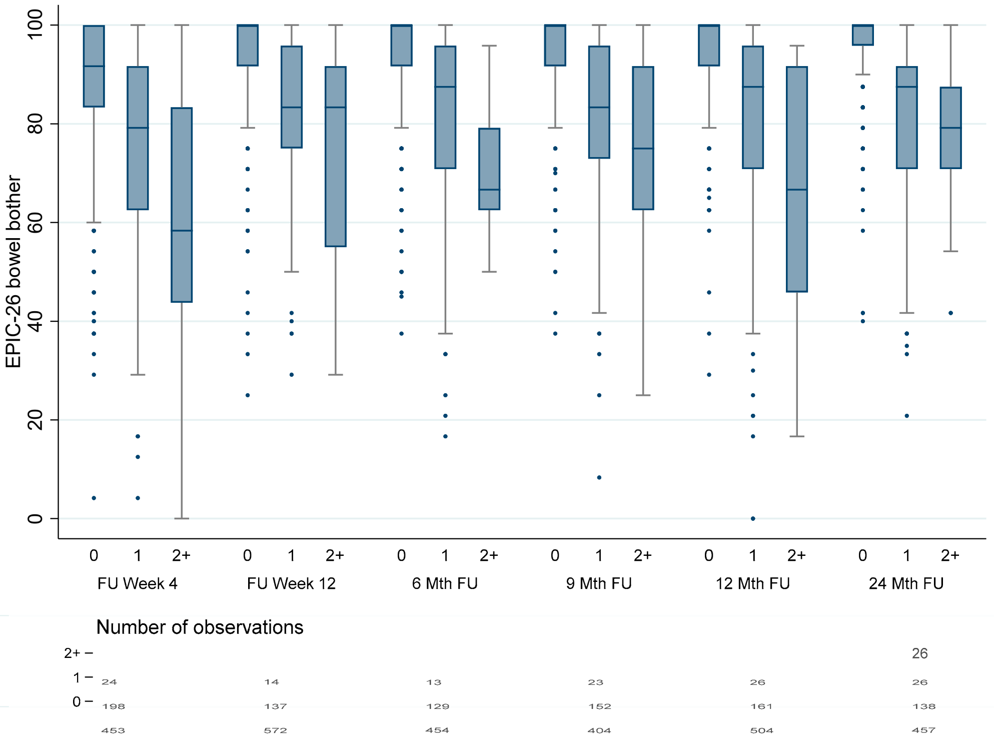

Figure S3: Association between EPIC-26 bowel sub-domain score and CTCAE GI grading across acute and late time periods (x axis).

Supplementary Table S11. Area under ROC curve (AUC) to assess models' ability to predict late GU and persistent late GU toxicity after SBRT and CRT.

Parameters	Stereotactic body radiotherapy		Conventional/moderately hypofractionated radiotherapy	
	AUC for predicting CTCAE G2+ GU late toxicity (6-24 months) (95% CI)	AUC for predicting CTCAE G2+ persistent late GU toxicity (95% CI)	AUC for predicting CTCAE G2+ GU late toxicity (6-24 months) (95% CI)	AUC for predicting CTCAE G2+ persistent late GU toxicity (95% CI)
**Univariable**				
Baseline G2+ GU toxicity	0.55 (0.52-0.58)	0.59 (0.54-0.63)	0.56 (0.52-0.60)	0.63 (0.55-0.72)
Baseline IPSS	0.63 (0.57-0.69)	0.63 (0.55-0.70)	0.63 (0.57-0.69)	0.65 (0.56-0.74)
Baseline EPIC-26 overall urinary bother score	0.63 (0.57-0.69)	0.67 (0.59-0.74)	0.64 (0.58-0.70)	0.65 (0.56-0.75)
Worst acute G2+ GU Toxicity	0.67 (0.62-0.72)	0.70 (0.64-0.76)	0.61 (0.55-0.66)	0.72 (0.63-0.82)
Persistent G2+ acute GU toxicity	0.62 (0.58-0.66)	0.70 (0.64-0.76)	0.62 (0.57-0.67)	0.77 (0.67-0.86)
Worst acute IPSS score	0.65 (0.59-0.71)	0.63 (0.56-0.71)	0.67 (0.60-0.73)	0.69 (0.59-0.78)
Worst acute EPIC-26 overall urinary bother	0.65 (0.59-0.70)	0.67 (0.60-0.74)	0.69 (0.63-0.76)	0.71 (0.63-0.80)
G2+ obstructive symptoms	0.57 (0.54-0.61)	0.62 (0.57-0.68)	0.56 (0.52-0.60)	0.69 (0.59-0.78)
G2+ irritative symptoms	0.63 (0.58-0.68)	0.65 (0.59-0.71)	0.57 (0.52-0.62)	0.58 (0.48-0.67)
G2+ pain/dysuria symptoms	0.56 (0.53-0.60)	0.57 (0.52-0.62)	0.51 (0.48-0.54)	0.52 (0.47-0.57)
G2+ incontinence symptoms	0.54 (0.51-0.56)	0.56 (0.52-0.60)	0.52 (0.50-0.55)	0.55 (0.49-0.61)
Prostate Volume	0.58 (0.52-0.64)	0.56 (0.49-0.64)	0.59 (0.52-0.66)	0.51 (0.39-0.63)
Age	0.53 (0.47-0.59)	0.52 (0.45-0.59)	0.51 (0.45-0.58)	0.53 (0.41-0.64)
Risk group	0.50 (0.47-0.53)	0.50 (0.47-0.54)	0.52 (0.49-0.55)	0.53 (0.49-0.57)
Baseline urinary medication	0.53 (0.49=0.57)	0.56 (0.51-0.62)	0.54 (0.48-0.59)	0.58 (0.49-0.68)
SBRT modality (CL)	0.58 (0.53-0.63)	0.57 (0.51-0.63)	-	-
Fiducial use (Yes)	0.54 (0.50-0.59)	0.55 (0.50-0.60)	0.54 (0.48-0.60)	0.65 (0.55-0.74)
**Multivariable analysis**				
Multivariable model A*	**0.73 (0.67-0.78)**	**-**	-	-
Multivariable model B **	**-**	**0.77 (0.70-0.83)**	-	-
Multivariable model C†	**-**	**-**	**0.66 (0.59-0.73)**	-
Multivariable model D‡	**-**	**-**	**-**	**0.77 (0.67-0.87)**

AUC = Area under curve. *Multivariable model for predicting late grade 2+ GU toxicity after SBRT. Parameters include baseline G2+ GU symptoms, worst acute grade 2+ GU toxicity, age, prostate volume, fiducial marker use and SBRT modality.**Multivariable model for predicting persistent late grade 2+ GU toxicity after SBRT. Parameters include baseline G2+ GU symptoms, worst acute grade 2+ GU toxicity, prostate volume, baseline urinary medication, fiducial marker use and SBRT modality. † Multivariable model for predicting late grade 2+ GU toxicity after CRT. Parameters include baseline G2+ GU symptoms, worst acute grade 2+ GU toxicity and prostate volume ‡ Multivariable model for predicting persistent late grade 2+ GU toxicity after CRT. Parameters include baseline G2+ GU symptoms, worst acute grade 2+ GU toxicity and fiducial marker use

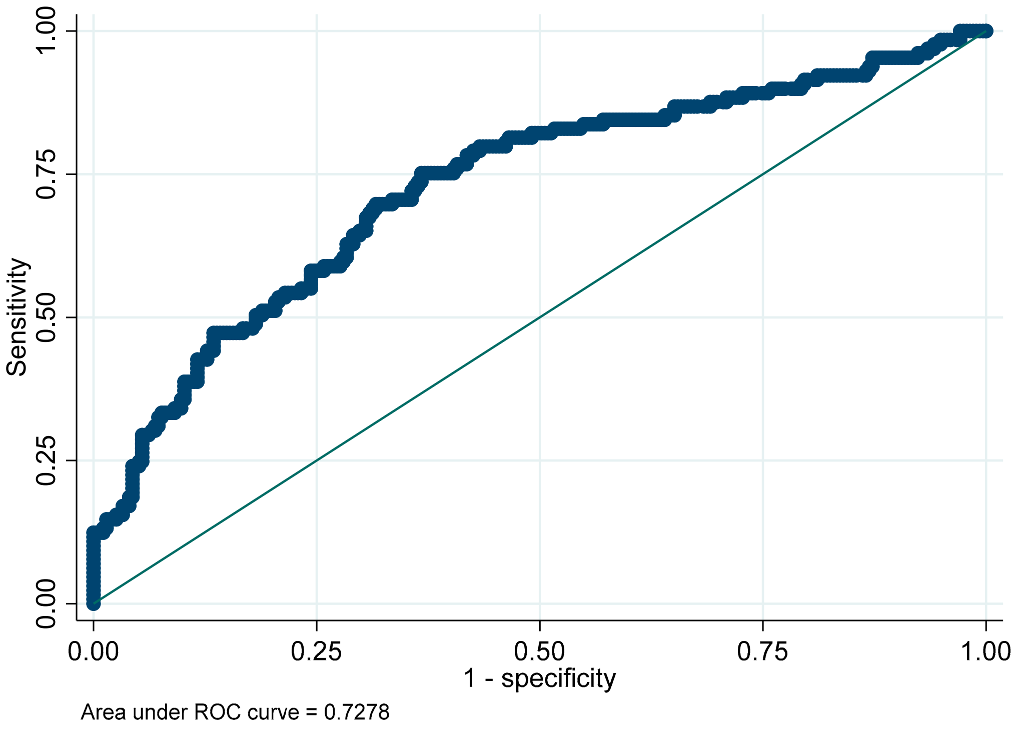

Figure S4: Receiver operating characteristic (ROC) curves for multivariable logistic regression predict (CTCAE G2+ late GU after SBRT). Parameters includes baseline G2+ GU symptoms, worst acute grade 2+ GU toxicity, age, prostate volume, fiducial marker use and SBRT modality)

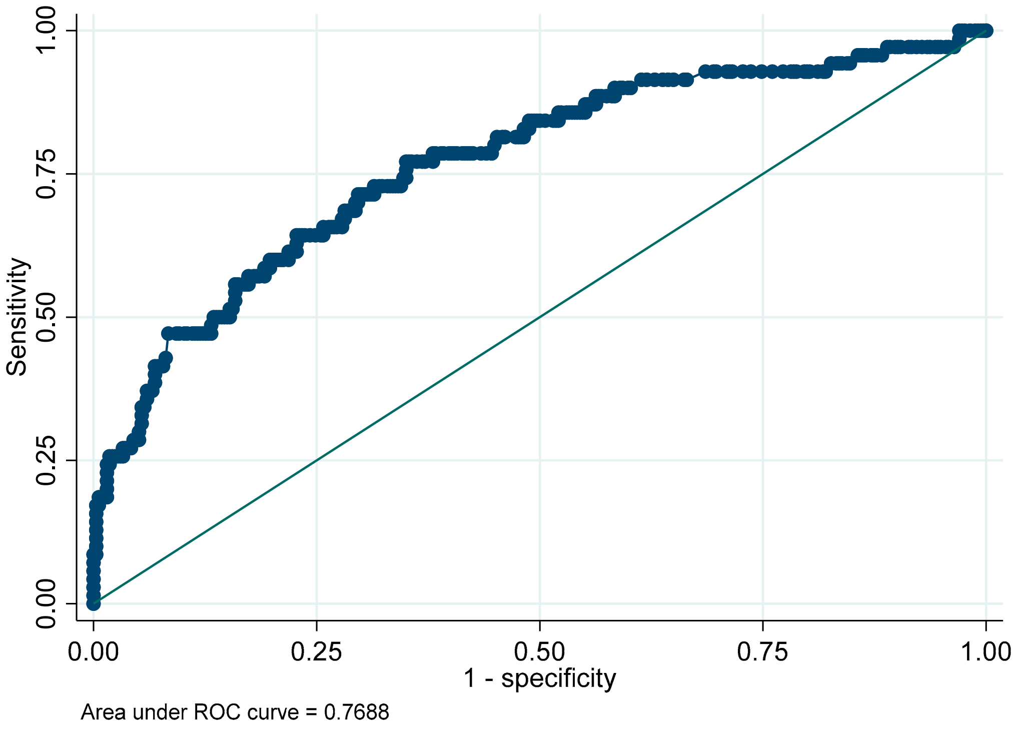

Figure S5: Receiver operating characteristic (ROC) curves for multivariable logistic regression (predict CTCAE G2+ persistent late GU after SBRT). (baseline G2+ GU symptoms, worst acute grade 2+ GU toxicity, prostate volume, baseline urinary medication, fiducial marker use and SBRT modality).

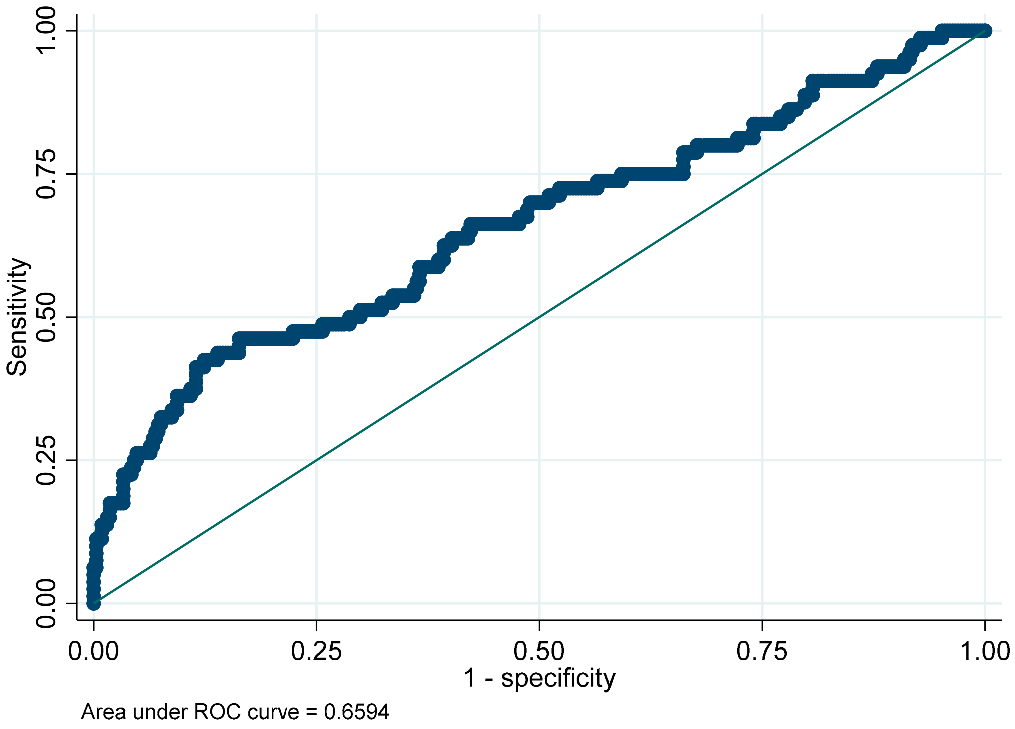

Figure S6: Receiver operating characteristic (ROC) curve for the multivariable logistic regression (predict G2+ late GU toxicity (6-24 months) after CRT). Parameters include baseline G2+ GU symptoms, worst acute grade 2+ GU toxicity, age, baseline urinary medication, risk group and prostate volume.

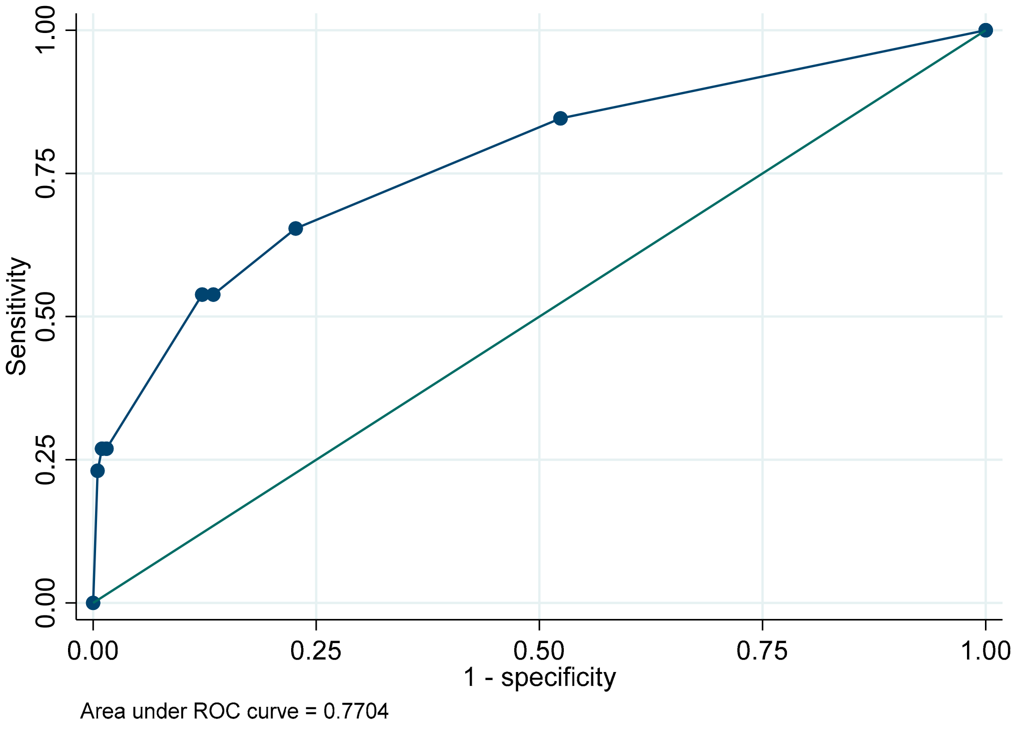

Figure S7: Receiver operating characteristic (ROC) curve for multivariable logistic regression (predict CTCAE G2+ persistent late GU after CRT). Parameters include baseline G2+ GU symptoms, worst acute grade 2+ GU toxicity and fiducial marker use.

Supplementary Table S12. Area under ROC curve (AUC) to assess models' ability to predict late GI and persistent late GI toxicity after SBRT and CRT.

Parameters	Stereotactic body radiotherapy		Conventional/moderately hypofractionated radiotherapy	
	AUC for predicting CTCAE G2+ GI late toxicity (6-24 months) (95% CI)	AUC for predicting CTCAE G2+ persistent late GI toxicity (95% CI)	AUC for predicting CTCAE G2+ GI late toxicity (6-24 months) (95% CI)	AUC for predicting CTCAE G2+ persistent late GI toxicity (95% CI)
**Univariable**				
EPIC-26 bowel sub-domain score (baseline)	0.58 (0.50-0.67)	0.59 (0.46-0.73)	0.56 (0.48-0.64)	0.60 (0.47-0.73)
Worst acute G2+ GI Toxicity	0.61 (0.54-0.68)	0.63 (0.53-0.74)	0.58 (0.52-0.64)	0.54 (0.45-0.63)
Persistent G2+ acute GI toxicity	0.53 (0.49-0.56)	0.55 (0.48-0.63)	0.53 (0.50-0.57)	0.52 (0.47-0.58)
Worst EPIC-26 bowel sub-domain score	0.59 (0.50-0.68)	0.66 (0.53-0.78)	0.57 (0.48-0.66)	0.64 (0.52-0.77)
G1+ rectal bleeding	0.57 (0.50-0.64)	0.61 (0.50-0.72)	0.55 (0.49-0.61)	0.56 (0.45-0.67)
G2+ bowel frequency	0.55 (0.50-0.60)	0.56 (0.48-0.64)	0.51 (0.49-0.54)	-
G1+ rectal pain	0.65 (0.57-0.72)	0.62 (0.51-0.73)	0.57 (0.51-0.63)	0.54 (0.48-0.60)
G2+ proctitis	0.54 (0.49-0.58)	0.57 (0.48-0.65)	0.52 (0.49-0.55)	-
Prostate Volume	0.50 (0.42-0.59)	0.49 (0.36-0.63)	0.53 (0.44-0.61)	0.58 (0.45-0.72)
Age	0.53 (0.44-0.62)	0.55 (0.43-0.67)	0.51 (0.43-0.59)	0.56 (0.42-0.69)
Risk group	0.53 (0.48-0.58)	0.50 (0.44-0.57)	0.55 (0.53-0.57)	-
SBRT modality (CL)	0.59 (0.52-0.66)	0.69 (0.62-0.77)	-	-
Fiducial use (Yes)	0.55 (0.48-0.62)	0.57 (0.47-0.68)	0.55 (0.48-0.62)	0.55 (0.44-0.67)
Multivariable model A*	**0.66 (0.57-0.75)**	**-**	-	-
Multivariable model B **	**-**	**-**	**0.64 (0.57-0.72)**	-

*Multivariable model for predicting late grade 2+ GI toxicity after SBRT. Parameters include worst grade 2+ acute toxicity, risk group, SBRT modality and fiducial use. **Multivariable model for predicting persistent late grade 2+ GI toxicity after CRT. Parameters include worst grade 2+ acute toxicity, risk group and fiducial use.

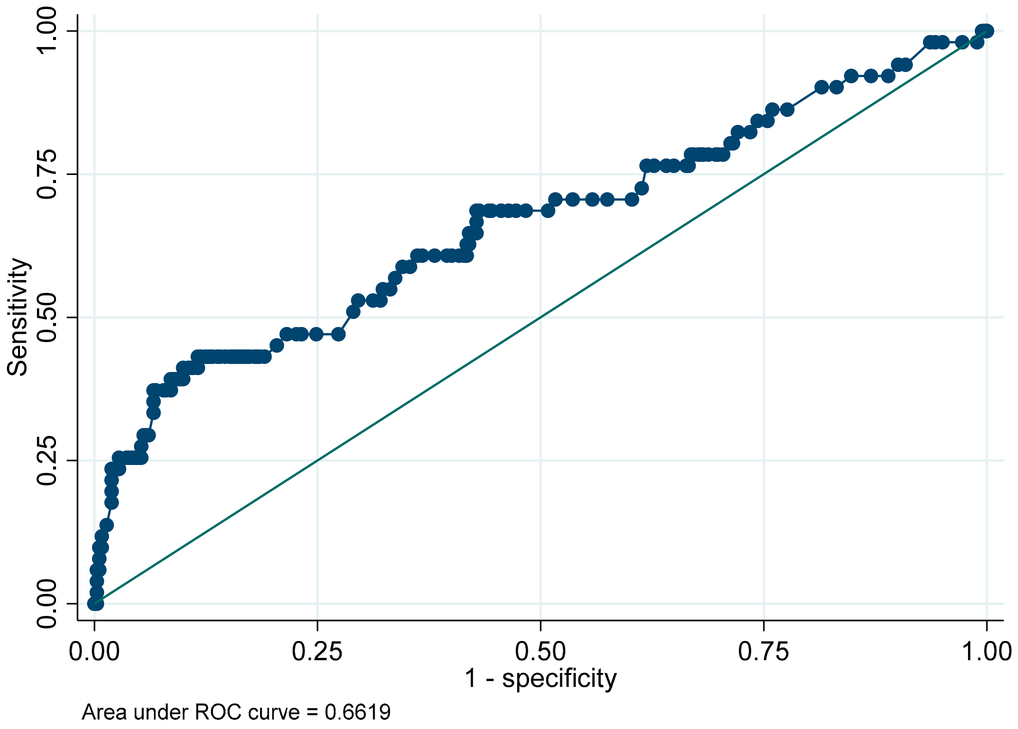

Figure S8: Receiver operating characteristic (ROC) curve for multivariable logistic regression (predict CTCAE G2+ late GI after SBRT). Parameters includes worst acute grade 2+ GI toxicity, risk group, fiducial use and SBRT modality.

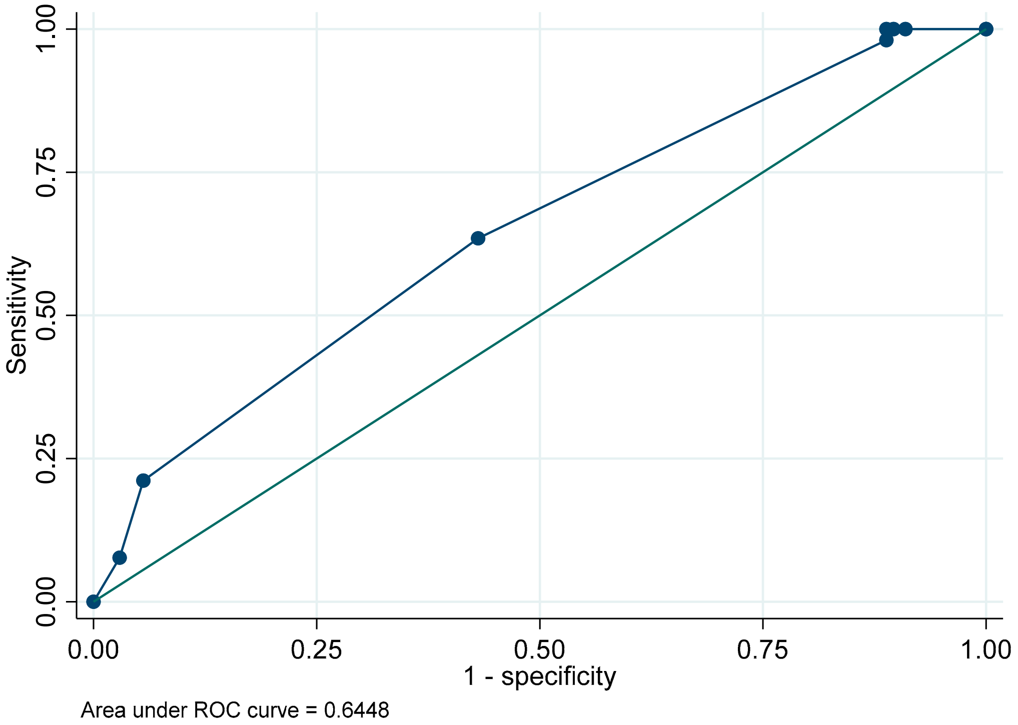

Figure S9: Receiver operating characteristic (ROC) curve for multivariable logistic regression (predict CTCAE G2+ late GI after CRT). Parameters includes worst acute grade 2+ GI toxicity, risk group and fiducial use.
